# Small cisterno-lumbar gradient of phosphorylated Tau protein in geriatric patients with suspected normal pressure hydrocephalus

**DOI:** 10.1186/s12987-016-0039-9

**Published:** 2016-08-31

**Authors:** Marija Djukic, Annette Spreer, Peter Lange, Stephanie Bunkowski, Jens Wiltfang, Roland Nau

**Affiliations:** 1 Department of Geriatrics, Evangelisches Krankenhaus Göttingen-Weende, Göttingen, Germany; 2 Institute of Neuropathology, University Medical Center Goettingen (UMG), Robert-Koch-Strasse 40, D-37075 Göttingen, Germany; 3 Department of Neurology, University Medical Center Goettingen (UMG), Göttingen, Germany; 4 Department of Neurology, University Medical Centre Mainz, Mainz, Germany; 5 Department of Psychiatry and Psychotherapy, University Medical Center Goettingen (UMG), Göttingen, Germany; 6 German Center for Neurodegenerative Diseases (DZNE), Georg August University Göttingen, Göttingen, Germany

**Keywords:** Cerebrospinal fluid, Amyloid beta 1–40, Amyloid beta 1–42, Tau protein, Phosphorylated Tau protein

## Abstract

**Background:**

The composition of the cerebrospinal fluid (CSF) is not homogeneous, and concentrations of proteins from different origins diverge among ventricular, cisternal and lumbar CSF fractions. Concentrations of blood-derived proteins increase and of brain-derived proteins decrease from ventricular to lumbar fractions. We studied whether the origin of the CSF portion analysed may affect results in CSF analysis for dementia.

**Methods:**

In 16 geriatric patients with suspected normal pressure hydrocephalus [age 82.5 (76/87) years; median (25th/75th percentile)] a lumbar spinal tap of 40 ml was performed. The CSF was sequentially collected in 8 fractions of 5 ml with the 1st fraction corresponding to lumbar CSF, the 8th to cisterna magna-near CSF. Fractions were analysed for total protein, albumin, Tau protein (Tau), phosphorylated Tau (pTau), Amyloid beta 1–42 (Aβ1–42), Amyloid beta 1–40 (Aβ1–40), and the Aβ1–42/Aβ1–40 ratio.

**Results:**

The concentrations of total protein and albumin increased from cisternal to lumbar fractions due to diffusion-related accumulation from blood to CSF with significantly higher concentrations in fraction 1 compared to fraction 8. The concentrations of Tau showed a non-significant trend towards decreased values in lumbar samples, and pTau was slightly, but significantly decreased in the lumbar fraction 1 [26.5 (22.5/35.0) pg/ml] compared to the cistern-near fraction 8 [27.0 (24.2/36.3) pg/ml] (p = 0.02, Wilcoxon signed rank test). Aβ1-42, Aβ1-40, and the Aβ1-42/Aβ1-40 ratio remained almost constant.

**Conclusions:**

According to the flow-related diverging dynamics of blood-derived and brain-derived proteins in CSF, the concentrations of Tau and pTau tended to be lower in lumbar compared to cisternal CSF fractions after a spinal tap of 40 ml. The differences reached statistical significance for pTau only. The small differences will not affect clinical interpretation of markers of dementia in the vast majority of cases.

**Electronic supplementary material:**

The online version of this article (doi:10.1186/s12987-016-0039-9) contains supplementary material, which is available to authorized users.

## Background

Normal pressure hydrocephalus (NPH) is characterized clinically by the triad of gait disturbance, bladder incontinence, and later dementia. Cerebral imaging shows ventricular dilation, whereas the subarachnoid space on both sides of the superior sagittal sinus is comparatively small [1]. Unlike in acute or subacute obstructive hydrocephalus, response to surgical shunt treatment in NPH is difficult to predict [2]. At autopsy, many of these patients display pathological findings suggesting neurodegenerative disease. Morphological findings of Alzheimer's dementia (AD) were detected in 30 to 75 % of patients with clinically diagnosed NPH where a biopsy was available suggesting a large subgroup of patients with overlapping clinical features of both AD and NPH [1–5]. A spinal tap of 40 ml is a relatively insensitive, minimally-invasive procedure to assess which patients may benefit from ventriculoperitoneal shunting.

Many compounds do not distribute homogeneously in the CSF spaces, but have ventriculo-lumbar or lumbo-ventricular gradients. Compounds originating in the brain (e.g., Tau protein, neuron-specific enolase, S-100 protein) generally have higher ventricular than lumbar CSF concentrations, whereas compounds originating from the meninges (e.g., β-trace protein and cystatin C) have higher lumbar than ventricular levels, and the concentrations of blood-derived proteins such as albumin increase from the ventricular to the lumbar CSF space [6–8]. In pathological conditions with decreased CSF flow or in central nervous system bacterial infections, where the CSF spaces can be, in part, obstructed by pus, the distribution of leukocytes and proteins can be particularly inhomogeneous, whereas the small molecule lactate is distributed homogeneously even in this condition [9]. Amyloid beta 1–42 (Aβ1–42, molecular mass 4514 Da), Amyloid beta 1–40 (Aβ1–40, molecular mass 4330 Da), Tau protein (Tau) and Tau protein phosphorylated at position 181 (pTau) (molecular mass 36800–45900 Da) are proteins originating from the brain and the myelon which help to discriminate AD from other types of dementia [4]. In a usual diagnostic lumbar puncture, approximately 5–10 ml of CSF is removed, and CSF analysis is performed on this fraction. Here, we collected 40 ml of CSF in fractions of 5 ml, to assess whether the markers are evenly distributed in these CSF fractions, or whether a ventriculo-lumbar concentration gradient can be also detected between the cistern-near and lumbar fractions, a situation which may affect the interpretation of these markers in the differential diagnosis of dementia.

## Methods

A diagnostic or therapeutic lumbar spinal tap of 40 ml was performed on 16 patients with suspected or documented normal pressure hydrocephalus [11 women, 5 men, age 82.5 (76/87) years; median (25th/75th percentile)]. All patients suffered from cognitive impairment. In 15 of the 16 patients an abnormal gait, and in 10 patients bladder incontinence were documented. All patients had an abnormal cranial computer tomography (CCT) scan with wide ventricles compared to the width of the sulci. Moreover, 14 patients had crowding of the gyri at the vertex with small sulci close to the superior sagittal sinus, and 14 patients had a corpus callosum angle below 90°. Four patients (including a patient who received three lumbar punctures) clearly improved after the spinal taps, and in three patients a small clinically non-significant improvement of either gait or cognition were noted. None of the patients studied had a ventriculoperitoneal shunt or external ventriculostomy. The treating physicians recommended ventriculoperitoneal shunting to the 4 patients with clear clinical improvement after the spinal tap. Because of their advanced age, these patients and/or their relatives refused surgery, as they feared the risks.

The CSF was sequentially collected in eight fractions of 5 ml in polypropylene tubes and was transported to the laboratory within 1 h for analysis. One set of CSF fractions was available from 15 patients. Three sets of CSF fractions were available from one patient with documented normal pressure hydrocephalus, who underwent repeated lumbar punctures as part of his therapy.

Albumin, immunoglobulins IgG, IgA and IgM were determined by nephelometry, and CSF leukocytes were counted manually in a Fuchs-Rosenthal chamber. Albumin and immunoglobulin CSF/serum quotients (IgG, IgA, IgM) were calculated in fraction 1. The CSF protein contents were measured by nephelometry using Gesamteiweiß UC Standard FS (DiaSys Diagnostic Systems, Holzheim, Germany). After centrifugation at 200×*g*, aliquots of each fraction were stored at −80 °C for the determination of markers of dementia, protein and albumin concentrations.

Tau protein (Tau), tau protein phosphorylated at threonine 181 (pTau), Amyloid beta 1–42 and Amyloid beta 1–40 (Aβ1–42 and Aβ1–40) were measured by commercially-available immunoassays (Innotest hTAU Ag, Innotest PHOSPHO-TAU 181P, Innotest β-AMYLOID 1–42, Fujirebio, Gent, Belgium; Amyloid-beta 1–40 CSF ELISA, IBL, Hamburg, Germany), and the Aβ1–42/Aβ1–40 ratio was calculated. The raw data were uploaded as Additional file 1 on Aug 6th, 2016.

Correlation of measured values between fraction 1 and fraction 8 of each individual patient was assessed by Spearman's rank correlation coefficient *r*_*S*_. The concentrations of the compounds measured in fractions 1 and 8 were compared by the two-tailed non-parametric paired Wilcoxon signed rank test using Gaph Pad Prism software, as most parameters were not normally distributed. *P* values <0.05 were considered statistically significant. The study was approved by the Ethics Committee of the Medical Faculty of the Georg-August University Göttingen, Germany, and each participant gave written informed consent to participate in this study.

## Results

The CSF leukocyte counts were normal in all patients studied, and the Reiber–Felgenhauer diagrams of albumin- and immunoglobulin-CSF/serum quotients (IgG, IgA, IgM) indicated absence of inflammation in all patients studied. Three patients had an elevated CSF-serum albumin ratio (10.2, 14.9 and 15.4 × 10^−3^) probably because of an impaired circulation of the CSF in the spinal canal: one of these patients had prior surgery because of stenosis of the lumbar spinal canal, one had a history of two fractures of lumbar vertebrae, and one suffered from lower back pain with hardening of muscles, suggesting degenerative spine disease. The concentrations of the parameters measured in fractions 1 and 8 of each individual patient were strongly correlated (*r*_*S*_ ≥ 0.81, *p* < 0.0001). The CSF protein contents were lower in the cistern-near fraction 8 than in the lumbar fraction 1 (*p* < 0.0001, Wilcoxon signed rank test) (Table 1). Albumin as a strictly blood-derived protein showed a rostrocaudal increase of concentration with significantly lower values in the cistern-near fraction 8 than in the lumbar fraction 1 (*p* < 0.0001, Wilcoxon signed rank test).

**Table 1:** Markers of dementia and protein concentrations in lumbar (fraction 1) and cistern-near cerebrospinal fluid (fraction 8) (n = 16 pairs of samples)

	CSF protein (mg/l)		Albumin (mg/l)		Tau (pg/ml)		PTau (pg/ml)		Aβ1–42 (pg/ml)		Aβ1–40 (pg/ml)		Aβ1–42/Aβ1–40	
Fraction	1	8	1	8	1	8	1	8	1	8	1	8	1	8
Median	433	353	253	202	188	210	26.5	27.0	985	955	12567	12576	0.077	0.075
25th percentile	309	240	177	138	145	151	22.5	24.2	765	783	11215	11045	0.065	0.065
75th percentile	513	427	288	234	280	265	35.0	36.3	1279	1191	15691	15465	0.088	0.087
Correlation between fraction 1 and 8 (*r*_*S*_)	0.95*		0.91*		0.94*		0.98*		0.97*		0.93*		0.81*	
Fraction 1 versus 8, *p* value; Wilcoxon signed rank test	<0.0001		<0.0001		NS		0.02		NS		NS		NS	

*NS* difference not significant

* *p* < 0.0001

pTau concentrations in the CSF fraction 8 were slightly, but significantly higher than in lumbar CSF (medians 27.0 versus 26.5 pg/ml, *p* = 0.02) indicating a small decrease of pTau from cisternal to lumbar CSF. Tau concentrations also were slightly higher in CSF fraction 8 than in lumbar CSF, the difference, however, failed to reach statistical significance (Table 1). No differences among fractions 8 and 1 were found for Aβ1–40, Aβ1–42 and the Aβ1–42/Aβ1–40 ratio. Only one patient had an elevated CSF Tau concentration (893 pg/ml in fraction 1 and 884 pg/ml in fraction 8). No patient had an elevated pTau (>61 pg/ml) or an abnormal CSF Aβ1–42 concentration (<450 pg/ml).

In the patient with 3 consecutive spinal taps in 11 months the intra-individual variation of the determined markers of neurodegenerative disease was low. Concentrations of Tau in the lumbar fraction 1 ranged from 148 to 176 pg/ml, of pTau from 25.5 to 28.0 pg/ml, of Aβ1–42 from 859 to 933 pg/ml, of Aβ1–40 from 8815 to 9731 pg/ml, and the Aβ1–42/Aβ1–40 ratio ranged from 0.088 to 0.106.

## Discussion

The total volume of CSF in adults is approximately 140 ml with a wide variation dependent on age, and volume of the brain and medulla spinalis in relation to the size of the cranial cavity and the spinal canal. The volume of the spinal subarachnoid fluid is 30–80 ml [6, 10], i.e., the first 5 ml fraction in our study represents lumbar CSF, whereas the 8th 5 ml fraction contains a large proportion of CSF from regions close to the cisterna magna and cisterna pontis.

In agreement with flow-related diverging dynamics of blood-derived and brain-derived proteins in CSF [7], we found an increase in the blood-derived protein albumin from cisternal to lumbar fractions and correspondingly an increase in total protein. In the analysis of markers of degenerative disorders, we found a small, statistically significant decrease in pTau from cisternal to lumbar fractions and a similar, yet not significant, decrease of Tau in fraction 1 (lumbar CSF) compared to fraction 8 (predominantly cisternal CSF), i.e. median pTau and Tau concentrations were higher in the cistern-near than in the lumbar CSF. In the patient receiving three spinal taps, the intra-individual variance between the different sampling points of pTau and Tau in lumbar CSF was higher (25.5–28.0 and 148–176 pg/ml, respectively) than the difference at group level between fraction 8 and 1 (27.0 versus 26.5 pg/ml and 210 versus 188 pg/ml). It has to be considered that these samples from one individual patient were drawn in an interval of 11 months. In two of these three samples from this patient, the pTau and Tau concentrations in cistern-near were higher than in lumbar CSF supporting our concept of a small cisterno-lumbar gradient of pTau and Tau. Similar rostro-caudal gradients have been found for α-synuclein and NSE in 5 patients with NPH [8]. In contrast to the small cisterno-lumbar concentration differences for pTau and Tau found in fractions 8 and 1 in the present study, the ventriculo-lumbar concentration ratios in NPH patients for Tau and pTau were previously found to be approximately five and twofold, respectively [11–13]. The existence of a ventriculo-lumbar Tau and pTau gradient was shown to depend on the diagnosis: it was large in NPH, but apparently absent in post-traumatic hydrocephalus [11]. The ventriculo-lumbar gradient observed previously and the cisterno-lumbar gradient observed by us suggests that Tau and pTau are mainly brain-derived, and that the fluids entering the spinal CSF from blood (interstitial fluid of the medulla spinalis and of the meninges) contain lower Tau and pTau concentrations than the interstitial fluid of the brain. This compares well with immunohistochemical findings demonstrating that in the spinal cord and the peripheral nervous system Tau immuno-reactivity is less abundant than in the brain [14]. Conversely, in previous studies lumbar Aβ1–40 and Aβ1–42 concentrations were slightly higher than the respective ventricular levels [12, 13]. In the present study, no clear gradients of Aβ1–40 and Aβ1–42 concentrations were observed, possibly as a consequence of their lower molecular mass compared to Tau proteins facilitating a more homogeneous distribution [9].

The median total protein content and the albumin concentrations were 1.23- and 1.25-fold higher, respectively, in fraction 1 than in fraction 8. This reflects a relevant influx of proteins from non-neuronal sources into the CSF in the spinal canal. In a previous study of 21 patients with NPH, 10 fractions of approximately 3 ml were drawn, and the albumin concentrations were 1.5-fold higher in fraction 1 than in fraction 10 [15]. Since the CSF flow dynamics are altered in NPH, these gradients of 1.25 or 1.5 in a spinal tap of 30–40 ml may not represent the gradients in healthy persons or patients with other diseases. Because high volumes of CSF can only be drawn with a clear clinical indication, it would be very difficult to obtain similar data in healthy adults or patients with other diseases. Patients with other forms of dementia, in particular those with Alzheimer`s disease, also develop brain atrophy (hydrocephalus ex vacuo) altering the dynamics of CSF flow, and many elderly patients suffer from degenerative spine disease impairing the circulation of the CSF in the spinal canal. For these reasons, we suggest that the cisterno-lumbar gradients of protein, albumin and markers of neurodegenerative diseases in the spinal canal may be similar in elderly patients with dementia irrespective of its cause. Lumbar (fraction 1) and cisternal (fraction 8) Tau, pTau, Aβ1–40 and Aβ1–42 concentrations and the Aβ1–40/Aβ1–42 ratios were closely correlated (Table 1) suggesting that these concentrations strongly depend on each other as a consequence of CSF bulk flow and diffusion.

## Conclusions

The rostro-caudal gradient of pTau and Tau in this and other studies on NPH suggests that both proteins are mainly brain-derived. This gradient, however, may not be present in all diseases. The differences between pTau and Tau concentrations in lumbar and cistern-near CSF were small, and the Aβ1–42 and Aβ1–40 concentrations and Aβ1–40/Aβ1–42 ratios in lumbar and cistern-near CSF were almost equal. Therefore, all fractions of CSF after a spinal tap of 40 ml appear to be equally suitable for the assessment of markers of dementia in CSF.

## Additional file

10.1186/s12987-016-0039-9 Dementia markers Spinal tap fraction 1 and 8.

## Abbreviations

Tau: Tau protein
pTau: phosphorylated Tau protein
Aβ1-42: Amyloid beta 1-42
Aβ1-40: Amyloid beta 1-40
Aβ1-42/Aβ1-40 ratio: ratio of Amyloid beta 1–42 and Amyloid beta 1–40
