# Supplementary material for: Small cisterno-lumbar gradient of phosphorylated Tau protein in geriatric patients with suspected normal pressure hydrocephalus
Source: Fluids Barriers CNS. 2016 Aug 31;13(1):15. doi: 10.1186/s12987-016-0039-9 (PMC5007695; doi:10.1186/s12987-016-0039-9)
Supplement: Supplementary file 1 — 10.1186/s12987-016-0039-9 Dementia markers Spinal tap fraction 1 and 8. [file 12987_2016_39_MOESM1_ESM.pdf]

Patient	Albumin fraction 1	Albumin fraction 8
1	282	215
2	242	122
3	237	199
4	165	145
5	263	222
6	263	204
7	138	135
8	92,9	73,5
9	670	420
10	213	190
11	263	196
12	755	446
13	290	238
14	141	115
15	298	258
16	225	209

Total protein fraction 1	Total protein fraction 8	Tau fraction1
442,6	359,5	141
387	221	158
387	221	893
267,9	212,4	248
503,3	462	291
515,8	422,7	158
294	294,9	345
159,3	121,4	132
1025,5	674,9	187
354,7	320,8	92
494,1	347,1	156
1128,9	728	214
534,7	428,7	218
248,9	200,3	94
479,1	405,7	189
423,4	397,5	351

Tau fraction 8	phospho-Tau fraction1	phospho-Tau fraction 8
145	23,2	25,4
169	25,5	26
884	55,5	57,7
199	22,6	23,9
270	41,5	40,8
220	25,3	26,4
341	36,1	37,1
127	28,3	30,1
232	22,4	21,4
95	19,1	19,2
195	27,4	27,6
234	27,9	33,8
248	31,8	32,5
96	17,4	17,2
184	22,3	25
373	45,1	44,1

A-beta1-42 fraction1	A-beta1-42 fraction 8	A-beta 1-40 fraction 1
771	766	10372
859	921	9731
707	776	13102
1299	1149	14713
1299	1242	16017
1217	1192	19021
632	616	13772
1147	1039	13172
1153	1188	16032
991	988	11302
1422	1375	11234
763	802	11302
791	832	10009
979	819	11208
652	591	12032
1519	1705	20302

A-beta 1-40 fraction 8	A-beta ratio fraction 1	A-beta ratio fraction 8
10323	0,07433475	0,07420324
8973	0,08827458	0,1026413
14723	0,05396123	0,05270665
15309	0,08828927	0,07505389
16732	0,08110133	0,07422902
18731	0,06398191	0,06363782
13021	0,04589021	0,0473082
13172	0,08707865	0,08753416
15517	0,07191867	0,07656119
11217	0,0876836	0,08808059
11372	0,12658	0,120911
11027	0,06751017	0,07273057
9932	0,07902888	0,08376963
12130	0,08734832	0,06751855
11099	0,05418883	0,05324804
21345	0,07482021	0,07987819
